# Interaction between Calpain-1 and HSP90: New Insights into the Regulation of Localization and Activity of the Protease

**DOI:** 10.1371/journal.pone.0116738

**Published:** 2015-01-09

**Authors:** Monica Averna, Roberta De Tullio, Marco Pedrazzi, Margherita Bavestrello, Matteo Pellegrini, Franca Salamino, Sandro Pontremoli, Edon Melloni

**Affiliations:** Department of Experimental Medicine (DIMES)—Biochemistry Section, and Center of Excellence for Biomedical Research (CEBR), University of Genoa, Viale Benedetto XV, 1–16132 Genoa, Italy

## Abstract

Here we demonstrate that heat shock protein 90 (HSP90) interacts with calpain-1, but not with calpain-2, and forms a discrete complex in which the protease maintains its catalytic activity, although with a lower affinity for Ca^2+^. Equilibrium gel distribution experiments show that this complex is composed by an equal number of molecules of each protein partner. Moreover, in resting cells, cytosolic calpain-1 is completely associated with HSP90. Since calpain-1, in association with HSP90, retains its proteolytic activity, and the chaperone is displaced by calpastatin also in the absence of Ca^2+^, the catalytic cleft of the protease is not involved in this association. Thus, calpain-1 can form two distinct complexes depending on the availability of calpastatin in the cytosol. The occurrence of a complex between HSP90 and calpain-1, in which the protease is still activable, can prevent the complete inhibition of the protease even in the presence of high calpastatin levels. We also demonstrate that in basal cell conditions HSP90 and calpain-1, but not calpain-2, are inserted in the multi-protein N-Methyl-D-Aspartate receptor (NMDAR) complex. The amount of calpain-1 at the NMDAR cluster is not modified in conditions of increased [Ca^2+^]_i_, and this resident protease is involved in the processing of NMDAR components. Finally, the amount of calpain-1 associated with NMDAR cluster is independent from Ca^2+^-mediated translocation. Our findings show that HSP90 plays an important role in maintaining a given and proper amount of calpain-1 at the functional sites.

## Introduction

Calpains are proteolytic enzymes that belong to a family of the Ca^2+^-dependent proteases, including the ubiquitously expressed calpain-1 and the calpain-2, which are distinguished by the optimal Ca^2+^ concentration for maximal activity. [1–3]. The activity of calpains can have either physiological or pathological roles depending on the extent and duration in [Ca^2+^]_i_ [4–6]. To express the physiological functions calpains require: 1) specific recognition of digestible substrates; 2) selective cellular localization; 3) proper mechanisms for regulating calpain activation and activity.

As up to now 200 proteins have been identified as calpain targets [3], specificity requirements of calpain cannot just be concerned with the nature of the substrate but rather with the translocation of the protease in close proximity to the appropriate target protein [7–10].

This hypothesis implies that selective processes could operate on the translocation and regulation of both the activation and activity of calpain. The mechanisms so far proposed involve variations in [Ca^2+^]_i_ and the interaction of calpain with its natural inhibitor calpastatin. This association prevents both translocation and expression of calpain activity [11, 12]. However, based on the present knowledge and on the fact that the amount of calpastatin largely exceeds that of calpain, it is currently still difficult to understand how calpain can translocate and express proteolytic activity. Yet, translocation of calpain could be involved in the localization of various calpain isoforms in mithocondria [7] as well as in nuclei [8, 9]. Moreover, calpain is able to specifically degrade members of protein complexes localized at the plasma membranes. These clusters contain both channels/receptors and enzymes that are required to regulate and elicit specific cell responses. For example, the ionotropic glutamate receptors NMDAR and AMPAR, the voltage gated sodium channel (NaCh) and the cystic fibrosis transmembrane conductance regulator (CFTR) are all calpain substrates [13–18]. The function of these channels is regulated by several proteins, specifically assembled in membrane clusters [19–22], that could represent a suitable model to establish how calpain can reach these structures and catalyze selective, limited, and controlled proteolysis. We have previously demonstrated that the reversible phosphorylation of calpastatin is responsible for changes in localization of the inhibitor [23]. This process capable of regulating the amount of calpastatin that interacts with calpain, essentially allows calpain to escape from calpastatin [23]. More recently it has been shown in neurons that calpain-1 and-2 undergo recruitment in different cell compartments where each one can apparently express different functions [14]. All these findings point to the existence of different mechanisms that leave calpain free from calpastatin restriction, and allow the translocation of the protease to selective functional sites.

In this paper we demonstrate for the first time that HSP90 specifically associates with calpain-1 and causes in the bound-calpain a decrease in the affinity for Ca^2+^. In resting JA3 cells which contain high levels of HSP90 [24, 25], cytosolic calpain-1 is associated with the chaperone. Moreover, since calpastatin competes with HSP90 for association to calpain-1, two different and discrete complexes can be present in cell cytosol. In the first one which contains calpain-1 and calpastatin, neither proteolytic activity nor translocation of the protease occurs. In the second one, in which calpain is associated with HSP90, the protease is only partially inhibited and trafficking of calpain-1 is not impaired.

Furthermore, we demonstrate that calpain-1 is also associated with the HSP90 at the NMDAR protein complex, and that the activation of this protease causes the functional proteolysis of specific components belonging to the channel cluster. Accordingly, we propose a new role for HSP90 in controlling the physiological amount and activity of calpain-1 at specific cellular localizations.

## Materials and Methods

### Materials

Aprotinin, leupeptin, calcium ionophore A23187, NMDA (*N*-methyl-D-aspartate), CI-1 (calpain inhibitor-1), Sephacryl S-300 HR, ferritin, aldolase, carbonic anhydrase, ovalbumin and IPTG (isopropyl β-D-1-thiogalactopyranoside) were purchased from Sigma-Aldrich. Pefabloc SC (4-(2-aminoethyl) benzenesulfonylfluoride, AEBSF) was obtained from Fluka. Foetal bovine serum (FBS), penicillin, streptomycin and L-glutamine were obtained from EuroClone. Geneticin was obtained from Invitrogen. ECL ADVANCE Detection System, PreScission Protease, and Protein G-Sepharose were obtained from GE Healthcare. Monoclonal anti-calpain-1 (calpain I, subunit p80) clone 15C10 and monoclonal anti-calpain-2 (Domain III/IV) clone 107–82 were obtained from Sigma-Aldrich. Monoclonal anti-HSP90 (clone 68), monoclonal anti-NMDAR2B (clone 13/NMDAR2B), and monoclonal anti-nNOS/NOS type I (clone 16) antibodies were obtained from BD Biosciences. Anti-NR1, CT monoclonal antibody was purchased from Millipore. Calpastatin was detected with the monoclonal antibody 35.23 [28] and monoclonal anti-calpastatin (Domain IV) clone 1F7E3D10 purchased from Calbiochem.

Human erythrocyte calpain (calpain-1) was isolated and assayed as reported in [3, 26]. HSP90 and calpain-2 were purified from rat brain as reported in [24, 27].

### Ethics Statement

Human erythrocytes and peripheral blood mononuclear cells (PBMC) were isolated from blood samples obtained from healthy donors (age range: 25÷60), that gave written informed consent prior to inclusion in the study, as well as permission to store the samples and to use them for research exclusively. The study protocol conforms to the provisions of the Declaration of Helsinki and of G. Gaslini Children Hospital, Genoa, Italy. The documentation related to participants consent is stored and recorded by G. Gaslini Children Hospital, Genoa Italy. Blood samples are collected and provided to the DIMES, Section of Biochemistry, anonymously under the supervision of Dr. L. Minicucci. The approval from the Ethics Committees is not required since our analysis were carried out on blood samples obtained from anonymous participants during their routine clinical examinations at the hospital and not for the purpose of this study.

The adult male rats (Milan strain 200–250 g) used for purification of HSP90 and calpain-2 were sacrificed by decapitation. The animals were housed at constant temperature (22±1°C) and relative humidity (50%) under a regular light-dark schedule (lights on 7 AM-7 PM). Food and water were freely available. Experimental procedures and animal care complied with the European Communities Council Directive of 24 November 1986 (86/609/EEC) and were approved by the Italian Ministry of Health in accordance with Decreto Ministeriale 116/1992 (protocol number 22698 of 17 September 2013). The related project dealt with the ethical and animal care aspects and was approved by the Committee set by the Ministry of Health at the National Institute of Health (Rome). Any effort was made to minimize the number of animals used and their suffering.

### Cell culture

Human leukemic T cell line (JA3), human neuroblastoma SK-N-BE cells (Interlab Cell Line Collection, ICLC, HTL96015, Italy) and rat pheochromocytoma PC12 cells, purchased from the American Type Culture Collection (A.T.C.C.) (Rockville, MD, USA) were cultured at 37°C (5% CO_2_) with RPMI 1640 growth medium containing 10% FCS, 10 U/ml penicillin, 100 μg/mL streptomycin and 4 mM L-glutamine. JA3-cast cells [28] were cultured in the same growth medium in the presence of 0.1 mg/mL geneticin.

### Preparation of recombinant calpastatin Type III

Mouse brain calpastatin Type III (GenBank AK029293) was obtained by amplification from single stranded cDNA generated from 5 µg total RNA. The amplicon was cloned into pGEX-6P-1 GST Expression Vector (GE Healthcare) using the forward primer Sn-EcoRI 5’-AAGAATTCATGAGTACCACAGAGACTAAGGCAATT and the reverse primer Asn-SalI 5’-AAAGTCGACGCTGAATTTCTATTCAGATACCCA. PCR reaction was cycled at an initial denaturating temperature of 98°C for 1 min followed by 35 cycles at 95°C for 30 s, 57°C annealing temperature for 30 s, and 72°C extension time for 2 min. A 5 min extension step at 72°C was performed after the last cycle of PCR. Library Efficiency DH5α Competent Cells (Invitrogen) were transformed with pGEX-6P-1/calpastatin Type III construct and ampicillin-resistant cells were selected. The sequence of cloned calpastatin Type III was confirmed by sequencing with CEQ 2000XL DNA analysis system (Beckman Coulter).

The GST fusion protein expression was induced in growing transformed DH5α cells by addition of 1 mM IPTG for 4 h at 37°C. Recombinant calpastatin Type III was purified to at least 95% homogeneity by GSH-agarose affinity chromatography followed by digestion with PreScission Protease in order to remove GST.

### HSP90 cloning and transfection

Human HSP90 (HSP90AB1, GenBank NM_007355.3) transcript was obtained by amplification of single stranded cDNA generated from 5 µg total RNA extracted from peripheral blood mononuclear cells. The amplicon was cloned into pcDNA3.1 (+) mammalian expression Vector (Invitrogen) using the forward primer Sn-NheI 5’-AAATTGCTAGCAAGATGCCTGAGGAAGTGC and reverse primer Asn-NotI 5’-TAAGCGGCCGCTCCTAACCTAATCGACTTCTTCCAT. PCR conditions were: a denaturation step for 1 min at 98°C; then 95°C for 30 s, 55°C for 30 s and 72°C for 2 min, for 35 cycles. A 5 min extension step at 72°C was performed after the last cycle of PCR. One Shot TOP10 Chemically Competent *E. Coli* (Invitrogen) were transformed with pcDNA3.1/HSP90 construct, the ampicillin-resistant cells were selected, and the vector was purified using HiSpeed Plasmid Maxi Kit (Qiagen). The sequence of cloned human HSP90 was confirmed by sequencing with CEQ 2000XL DNA analysis system (Beckman Coulter).

JA3-cast cells (250 000/well) were transfected with pcDNA3.1/HSP90 vector (2.5 μg) using 3 μL of DMRIE-C (Invitrogen) following the manufacturer’s instructions. At 40 h post-transfection, cells were harvested and processed for immunoprecipitation experiments or confocal microscopy analysis.

### Confocal microscopy

JA3, JA3-cast or JA3-cast transfected with HSP90 (JA3-cast-HSP90) cells (2 × 10^6^) were collected by centrifugation at 300 ***g*** for 10 min and washed three times with PBS. Cells were fixed and permeabilized with Triton/paraformaldehyde method [29]. Calpastatin was detected using the anti-calpastatin mAb 35.23 [29] as primary antibody. Chicken anti-(mouse IgG) Alexa fluor 488-conjugate (Life Technologies) was used as secondary antibody. The excitation/emission wavelengths were 488/522 nm. Images were collected using a Bio-Rad MRC1024 confocal microscopy, with a 60× Plan Apo objective with numerical aperture 1.4. The cytosolic fluorescence intensity in each collected image was quantified using LaserPix software (Bio-Rad) and followed the procedure described in [30].

### Immunoprecipitation and Immunoblotting

Aliquots (1.5 µg) of purified calpain-1- or-2 were incubated with different amounts of purified HSP90 in the presence or absence of different amounts of calpastatin Type III. The mixtures were immobilized to Protein G-Sepharose resin using monoclonal anti-HSP90 antibody (1 µg) in 100 µL (final volume) of 50 mM sodium borate buffer (pH 7.5) containing 1 mM EDTA, following a previously reported procedure [24]. After incubation, the immunoprecipitated material was eluted with 30 µL of SDS-PAGE loading buffer [31], heated for 5 min at 95°C, and submitted to 8% SDS-PAGE. Proteins were blotted onto a nitrocellulose membrane (Bio-Rad) and probed with anti-calpain-1 or anti-calpain-2 antibodies. The immunoreactive bands were developed with an ECL detection system, detected with a Bio-Rad Chemi Doc XRS apparatus, and quantified using the Quantity One software, release 4.6.1 (Bio-Rad). Alternatively, HSP90 (3 µg) was incubated with different amounts of calpain-1 and the immunoprecipitation was performed using monoclonal anti-calpain-1 (1 µg) as described above. Co-immunoprecipitated HSP90 was analysed and quantified by immunoblotting.

JA3, JA3-cast or JA3-cast-HSP90 cells (5 × 10^5^) were lysed in 50 µL of 50 mM sodium borate buffer (pH 7.5) containing 1 mM EDTA, 10 µg/mL aprotinin, 100 µg/mL leupeptin, and 2 mM Pefabloc SC by three freeze-thaw cycles and briefly sonicated. Cell cytosolic fraction was obtained by centrifugation at 100 000 ***g*** for 15 min at 4°C and pre-treated with protein G-Sepharose. Immunoprecipitation was carried out with 0.5 μg of monoclonal anti-HSP90 antibody. Proteins (input, bound to HSP90, and output) were separated by 8% SDS-PAGE and subjected to immunoblotting.

SK-N-BE cells (10^7^), PBMC (10^7^) isolated as previously described [32], PC12 cells (10^6^) and JA3 cells (10^7^) were lysed as described above and the membrane fraction was washed in 50 mM sodium borate buffer (pH 7.5) containing 0.1 mM EDTA and solubilized in 50 mM sodium borate buffer (pH 9.0), containing 0.1 mM EDTA and 1% sodium deoxycholate, at 37°C for 60 min. After centrifugation at 100 000 ***g*** for 30 min at 4°C, the pH was adjusted to pH 8.0, and Triton X-100 was added to a final concentration of 0.1%. The detergent-soluble portion was dialyzed against IP buffer [50 mM sodium borate buffer (pH 7.5), 0.1 mM EDTA, 0.1% Triton X-100] by diafiltration using centrifugal filter devices (10 kDa cut-off) Amicon Ultra-4 (Millipore). Before immunoprecipitation procedure, samples were pre-cleared for 60 min at 4°C using 30 µl of Protein G-sepharose diluted 1:1 with IP buffer. Immunoprecipitation was carried out using 1 µg of anti-NR1 antibody. Proteins were separated by 8% SDS-PAGE and subjected to immmunoblotting. The immunoreactive bands obtained by using the specific mAbs (indicated elsewhere) were quantified as described above.

### Equilibrium distribution experiments in Sephacryl S-300 HR

Equilibrium gel distribution (gel penetration) experiments were carried out according to the procedure described by Ackers [33] and modified by Fahien and Smith [34] and MacGregoret al. [35]. Briefly, protein samples (indicated elsewhere) were diluted in 0.25 mL of 50 mM sodium borate buffer (pH 7.5) containing 1 mM EDTA, 0.5 mM 2-mercaptoethanol and 0.15 M NaCl (buffer A) and added to 0.25 mL of packed Sephacryl S-300 HR previously equilibrated with buffer A. The mixtures were rotated end-over-end for 2 h at 4°C and finally the resin was packed. The distribution coefficient, that indicates the accessibility of the protein molecules to the gel bed internal space, was determined analysing aliquots (0.1 mL) of the clear aqueous phase as described in [33–35].

## Results

### Interaction of calpain-1 with HSP90

**Figure 1:**
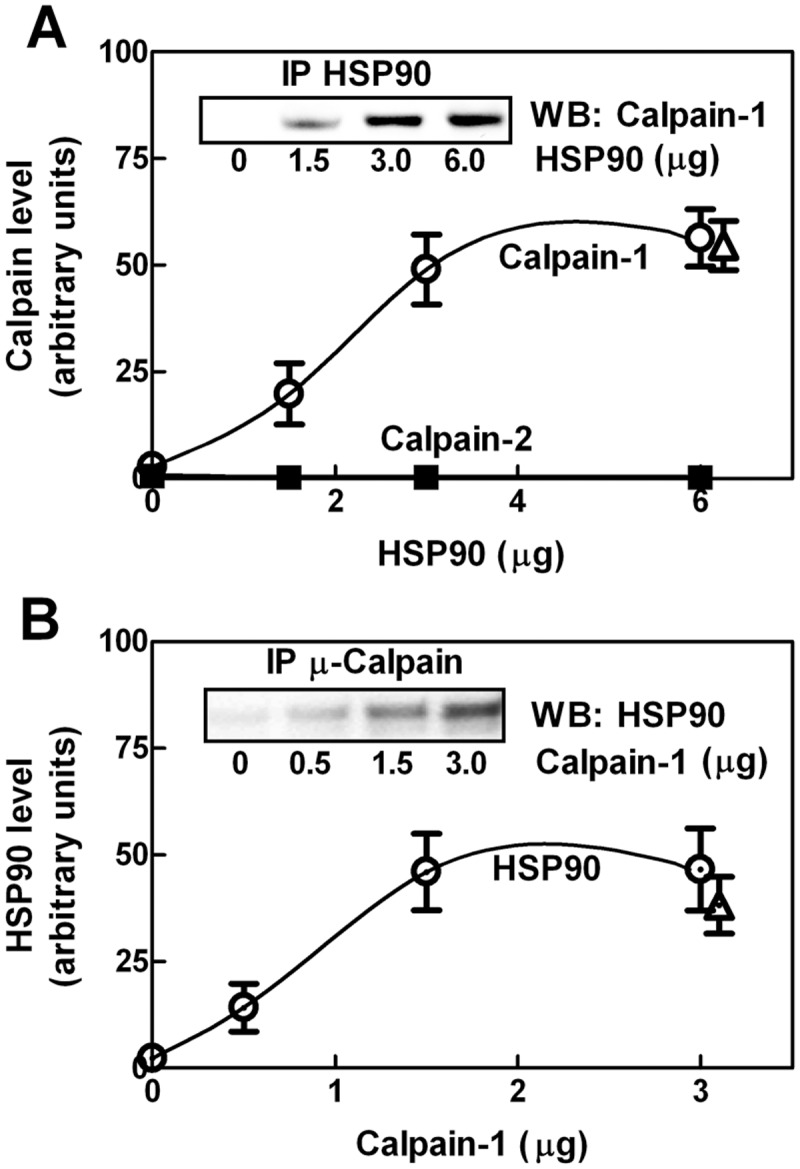
Interaction between calpain and HSP90. (A) Purified calpain-1 or-2 (1.5 µg) was incubated for 1 hour at 25°C with the indicated amounts of purified HSP90. Immunoprecipitation was carried out using 1 µg of anti-HSP90 antibody. Immunoprecipitated material (IP HSP90) was submitted to SDS-PAGE and analysed by immunoblotting using monoclonal anti-calpain-1 (○) or anti-calpain-2 (■). Inset: a representative blot for calpain-1 is shown. (B) Alternatively, HSP90 (3 µg) was incubated for 1 hour at 25°C with the indicated amounts of purified calpain-1. Immunoprecipitated material (IP Calpain-1), obtained using 1 µg of monoclonal anti-calpain-1, was submitted to SDS-PAGE and analysed by immunoblotting using monoclonal anti-HSP90. Inset: a representative blot is shown. Immunoprecipitations were also performed in the presence of 1 mM CaCl_2_ and 100 µg/mL leupeptin (Δ). Each point corresponds to the arithmetic mean ± SD of five different experiments.

Immunoprecipitation experiments (Fig. 1A and B) revealed that calpain-1 can associate to HSP90 in a saturable fashion. The interaction between the two proteins seems specific as no complex with HSP90 was detected when calpain-1 was replaced by calpain-2. The addition to the immunoprecipitation mixture of Ca^2+^ ions (Fig. 1A and B, triangles), known to promote a significant conformational change in calpain molecule, had no effect on the association between the two proteins. Even when we performed the same experiments in the presence of a calpain substrate (such as human globin) or of an indigestible protein (data not shown), we only observed a complex between calpain-1 and HSP90.

Thus, both catalytic cleft and the Ca^2+^-binding sites seem not directly involved in the interaction between HSP90 and calpain-1. By comparing the data shown in Fig. 1A and B, it can be seen that the plateau was reached in both conditions at 1:1 molar ratio chaperone to calpain, suggesting the formation of a discrete complex containing an equal number of both molecules.

**Figure 2:**
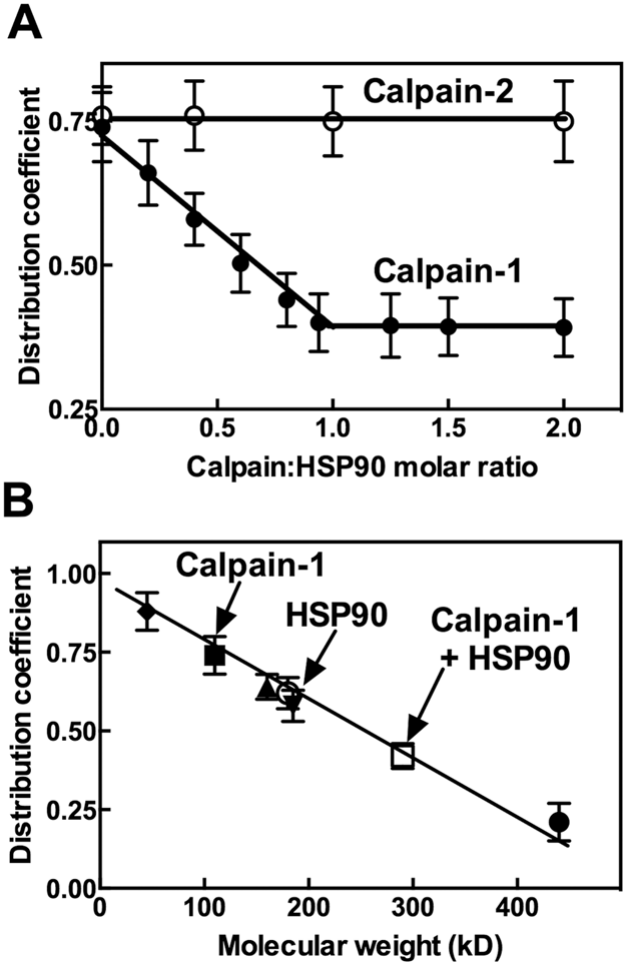
Molecular properties of the HSP90-calpain-1 complex. (A) HSP90, calpain-1 and calpain-2 were purified as reported in [24, 26, 27]. Calpain-1 or calpain-2 (0.5 μg, corresponding to 4.5 pmoles) were diluted in 0.25 mL of buffer A and added to 0.25 mL of packed Sephacryl S-300 HR (see Methods). HSP90 was also added to the mixtures in amounts (from 0 to 1.65 µg) corresponding to the indicated HSP90:calpains molar ratios. The suspensions were rotated end-over-end for 2 h at 4°C. Resin was then packed and aliquots (0.1 mL) of the clear supernatant were recovered and calpain activity was assayed [26]. Distribution coefficient of calpain was calculated as described in [33–35]. Each point corresponds to the arithmetic mean ± SD of two different experiments. (B) Distribution coefficient-molecular weight calibration curve was determined using the indicated standard proteins: ferritin (●), aldolase (▲), anhydrase carbonic (▼) and ovalbumin (♦). The proteins (10 pmoles) were added to Sephacryl S-300 HR as in (A) and the distribution coefficients were calculated evaluating the protein concentration, with the Bradford method, in the clear supernatants as described in [33–35]. The distribution coefficient of calpain-1 (■) and 1:1 HSP90-calpain-1 complex (□) was evaluated as in (A). The distribution coefficient of HSP90 (○) was determined as indicated for the standard proteins. Each point corresponds to the arithmetic mean ± SD of two different experiments.

To better characterize the HSP90-calpain-1 interaction, equilibrium gel distribution experiments [33–35] were carried out. As shown in Fig. 2A the distribution coefficient of calpain-1 in Sephacryl S-300 HR progressively decreased as a function of the amount of HSP90 added until a 1:1 molar ratio between the two proteins was reached. Higher levels of HSP90 did not promote further decrease in the distribution coefficient of calpain-1. Conversely, the distribution coefficient of calpain-2 was unaffected by the additions of HSP90, confirming the selectivity of the chaperone for calpain-1.

The formation of a 1:1 HSP90-calpain-1 complex has been confirmed by the calibration curve reported in Fig. 2B showing that this complex had a molecular mass of approximately 300 kD, corresponding to the MW sum of the two proteins.

### Effect of HSP90 on calpain-1 catalytic properties

**Figure 3:**
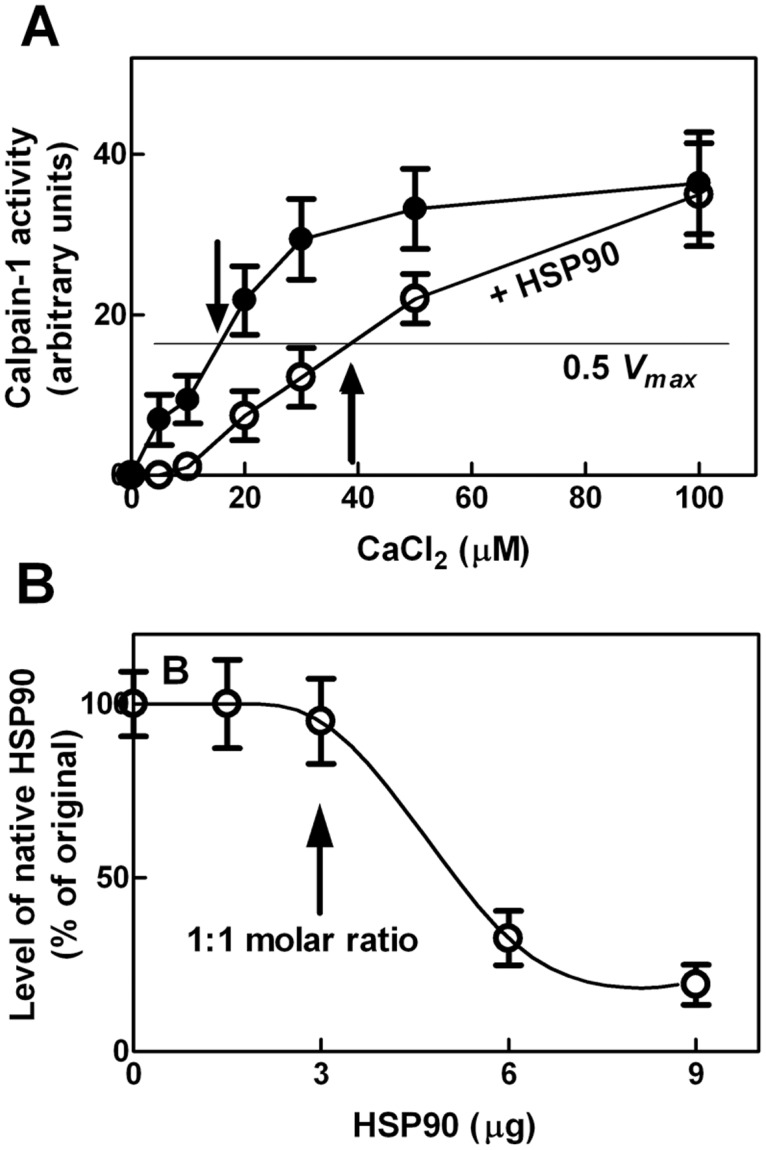
Calpain activity in the presence of HSP90. (A) Calpain-1 activity was assayed at the indicated CaCl_2_ concentrations in the absence (●) or presence (○) of purified HSP90 in a 1:1 molar ratio with calpain-1. The arrows indicate the 0.5 *Vmax* value of each condition. Data are the arithmetic mean ± SD of five different experiments. (B) The indicated amounts of purified HSP90 were incubated (100 µL final volume) with calpain-1 (1.5 µg) in 50 mM sodium borate buffer (pH 7.5), containing 0.1 mM EDTA, for 30 min at 37°C in the presence of 1 mM CaCl_2_. After incubation, the samples were suspended in SDS/PAGE loading buffer and aliquots (30 µL) were submitted to SDS/PAGE and analysed by immunoblotting using anti-HSP90 antibody. The arrow indicates the point corresponding to 1:1 molar ratio between HSP90 and calpain-1. Each point corresponds to the arithmetic mean ± SD of five different experiments.

As the association of the HSP90 with calpain-1 does not involve the catalytic region of the protease, we explored if the binding of calpain to HSP90 could affect the protease activity (Fig. 3A). Incubation of calpain-1 at a molar ratio of 1:1 with HSP90, at increasing Ca^2+^ concentrations, resulted in a significant decrease of calpain proteolytic activity at [Ca^2+^] up to 40 µM. At higher [Ca^2+^] when calpain expressed 100% activity, the inhibiting effect of HSP90 was no more detectable. This observation is consistent with a decrease in the affinity of calpain-1 for Ca^2+^ ions as, in the presence of HSP90, the [Ca^2+^] required to promote 50% activity of calpain-1 was increased from 16–18 µM to 40–45 µM. However, as it is known that HSP90 is a calpain substrate [24, 36], it is difficult to explain how the chaperone could at the same time associate with calpain-1 and undergo to proteolytic digestion by calpain-1 itself. We then mixed a fixed amount of calpain-1 with increasing concentrations of HSP90 (HSP90:calpain-1 molar ratios 0.5, 1, 2, 3) and monitored the chaperone digestion. As shown in Fig. 3B we detected HSP90 degradation only when the molar amount of the chaperone exceeded that of calpain-1. These data indicate that HSP90 binds to calpain-1 in a site distinct from the active cleft, leaving this site accessible to free HSP90 molecules.

### Interplay between HSP90 and calpastatin for binding to calpain-1

**Figure 4:**
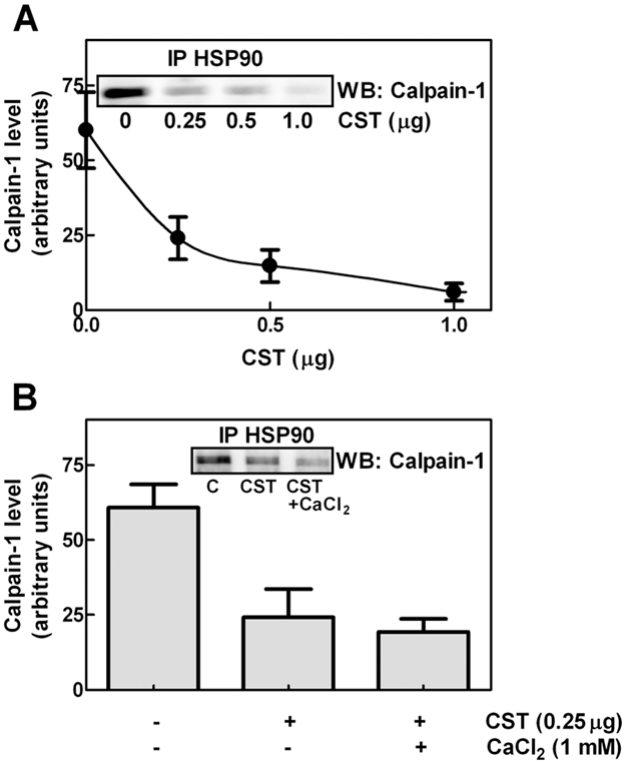
Effect of calpastatin on the interaction between calpain-1 and HSP90. (A) HSP90 and calpain-1 were purified as reported in [24, 26], recombinant calpastatin type III was purified as described in Methods. Calpain-1 (1.5 µg) was incubated for 1 hour at 25°C with 3 µg of HSP90 in the presence of the indicated amounts of recombinant calpastatin type III (CST). The immunoprecipitation was carried out using 1 µg of anti-HSP90 antibody and the immunoprecipitated material (IP HSP90) was analysed by immunoblotting using monoclonal anti-calpain-1. (B) The immunoprecipitation described in (A) was also performed in the presence of the indicated additions. Leupeptin (100 µg/mL) was added in order to avoid calpain activation in the presence of 1 mM CaCl_2_. Immunoreactive bands were quantified and the values are reported as the arithmetic mean ± SD of five different experiments. Insets: representative blots are shown.

It is well known that calpastatin, the natural inhibitor of calpain, can bind to the protease at the active cleft [11]. We have previously reported [37] that the calpastatin forms containing the N-terminal L-domain can associate with calpain-1 also in the absence of calcium. In these conditions calpain is in its inactive conformation and the catalytic cleft remains inaccessible. We then established whether in the absence of Ca^2+^, calpastatin and HSP90 can compete for calpain through interaction at the same region. Fixed amounts of calpain-1 and HSP90 in a 1:1 molar ratio were mixed with increasing concentrations of calpastatin Type III that contains the L-domain. As shown in Fig. 4A, the calpain-1 bound to HSP90, detected by immunoprecipitation, reduced proportionally to the amounts of calpastatin used. Since addition of 1 mM Ca^2+^ did not significantly affect the binding competition between calpastatin and HSP90 (Fig. 4B), we suggest that the accessibility of the catalytic cleft is not important for this protein-protein interaction.

### Interplay among HSP90, calpastatin, and calpain in cytosol of JA3 cells

**Figure 5:**
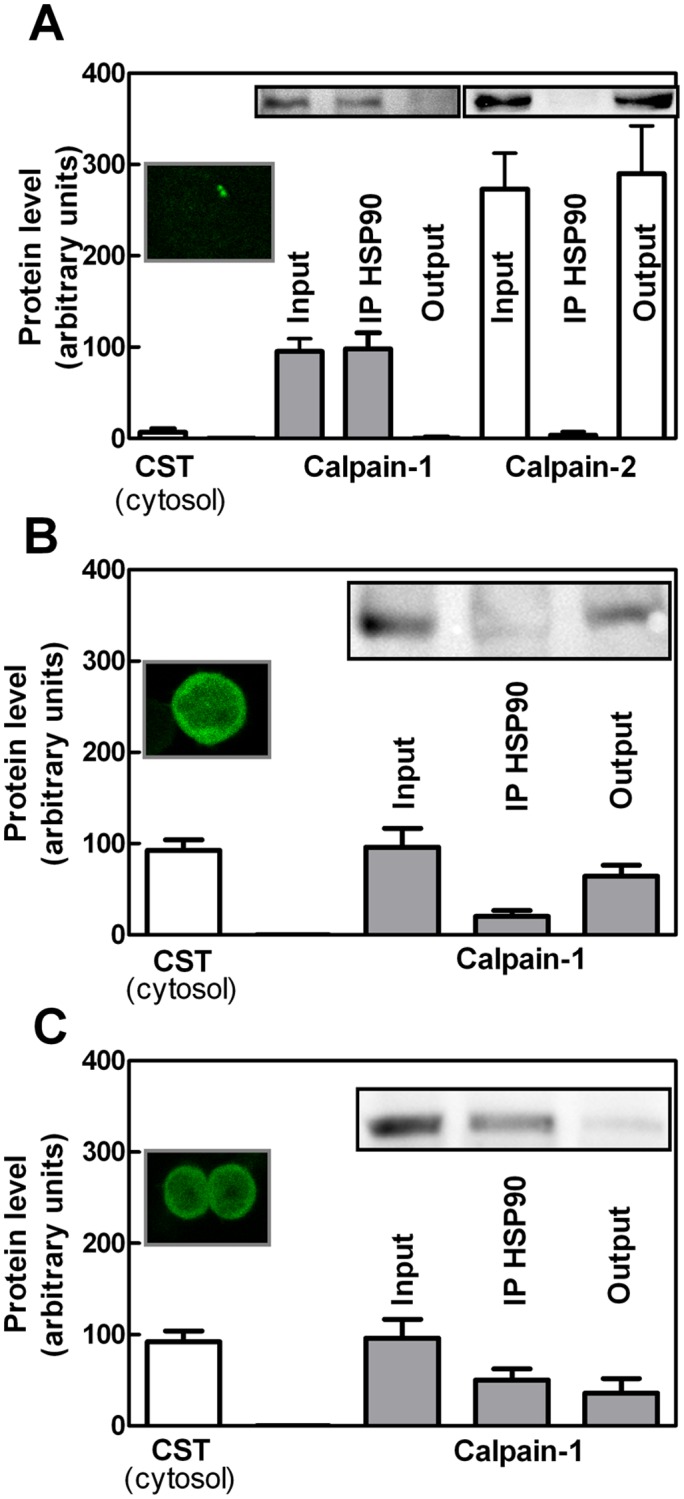
Interaction between calpain-1 and HSP90 in JA3, JA3-cast, and JA3-cast-HSP90 cells. (A) JA3, (B) JA3-cast, and (C) JA3-cast-HSP90 cells were analysed by confocal microscopy or lysed to perform immunoprecipitation using 0.5 µg of anti-HSP90 antibody (see Methods). Cytosolic fluorescence of calpastatin (CST) was quantified as described in [30]. A representative micrograph of 20 cells analysed is shown. The immunoreactive bands corresponding to calpain-1 or-2 present in the starting material (Input), in the HSP90-bound material (IP HSP90), and in the unbound material (Output) were quantified and the values are reported as the arithmetic mean ± SD of five different experiments. Insets: representative blots are shown.

We then explored whether the association of calpain-1 with HSP90 or calpastatin occurs also in intact cells. To this purpose we used JA3 cells as a model because they naturally express high levels of HSP90 [24] and because we have previously selected a clone (JA3-cast) stably overexpressing a calpastatin form that contains the L-domain [28]. As shown in Fig. 5A (micrograph), in resting JA3 cells, calpastatin is confined in perinuclear aggregates [29]. Instead we observed by immunoprecipitation that, soluble calpain-1 was entirely complexed with HSP90, as demonstrated by the absence of the protease in the output (Fig. 5A). Thereby, in basal conditions calpain-1 associates exclusively with HSP90 in a highly specific manner. In fact, calpain-2 was never recovered in association with HSP90 (Fig. 5A). In order to verify whether calpastatin and HSP90 compete with each other also in intact cells, we used JA3-cast cells [28] in which over-expressed calpastatin is diffused into the cytosol as a free form (Fig. 5B, micrograph). In these cells, the amount of calpain-1 complexed with HSP90 was 70–80% reduced and the remaining protease was recovered in the HSP90 unbound material. The competition between HSP90 and calpastatin for binding to calpain-1 was further supported by transfecting the JA3-cast cells with HSP90. Under these conditions of transient over-expression of HSP90 (Fig. 5C), we observed a significant increase (p<0.05 according to *t* test) in the amount of calpain-1 associated with the chaperone (Fig. 5C).

### Presence of calpain-1 and HSP90 in NMDAR clusters of different cell types

**Figure 6:**
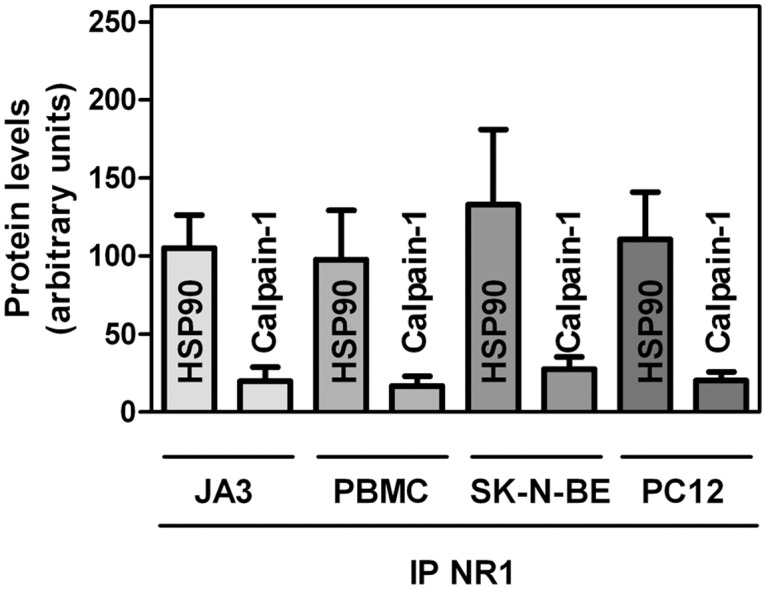
Presence of calpain-1 and HSP90 in NR1 immunoprecipitates from different cell types. PBMC, JA3, SK-N-BE, and PC12 cells were lysed to perform immunoprecipitation with 1 µg of anti-NR1. The immunoprecipitated material (IP NR1) was analysed by immunoblotting. Immunoreactive bands corresponding to HSP90 and calpain-1 were quantified and the values are reported as the arithmetic mean ± SD of two different experiments.

As we have observed that resting cells contain in cytosol high amounts of HSP90-calpain-1 complex, we investigated whether these two proteins can be detected at well defined cellular localizations, in which some calpain-1 targets are present. One of these proteins is NMDAR, a Ca^2+^-permeable NMDA-type glutamate receptor [38] known to undergo a selective processing by calpain [39–41]. For this purpose, we performed NR1 immunoprecipitation using isolated membranes from several cell types. Specifically, resting JA3 cells, human peripheral blood mononuclear cells (PBMC), rat pheochromocytoma PC12 cells, and human neuroblastoma SK-N-BE cells, all expressing NMDAR [42–47] have been used. As shown in Fig. 6, we observed detectable amounts of calpain-1 and HSP90 associated to NMDAR in all the cell types analysed. These results suggest that both HSP90 and calpain-1 are constitutively present in the NMDAR cluster and could be possibly involved in the physiological function of this protein complex.

### Role of HSP90 in calpain-1 activation at the NMDAR cluster

**Figure 7:**
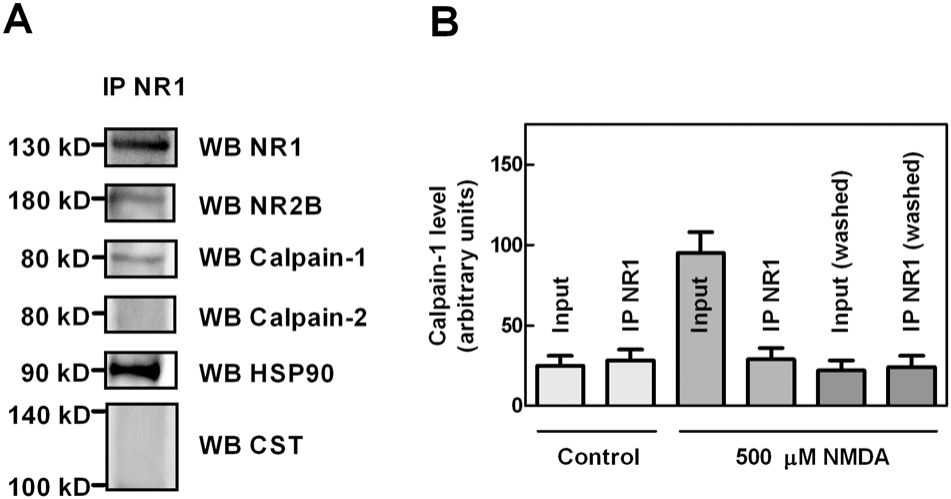
Characterization of calpain-1 insertion in NMDAR cluster. (A) SK-N-BE cells were lysed to perform immunoprecipitation with 1 µg of anti-NR1. The immunoprecipitated material (IP NR1) was analysed by immunoblotting using the indicated antibodies. Relevant lanes of representative blots are shown. (B) SK-N-BE cells, untreated (Control) or exposed to 500 µM NMDA for 30 min, were lysed and cell lysate was divided in two equal aliquots. The first aliquot was centrifuged and the resulting pellet was solubilised in 1% sodium deoxycholate. An aliquot of the solubilised membranes (10 μL) was blocked by adding 6× SDS/PAGE loading buffer (Input) and the remaining membrane fraction was submitted to immunoprecipitation using 1 µg of anti-NR1 antibody. The input and the immunoprecipitated material (IP NR1) were analysed by immunoblotting using the anti-calpain-1 antibody. The second aliquot of cell lysate was washed once with 50 mM sodium borate buffer (pH 7.5), containing 0.1 mM EDTA and 0.15 M NaCl, and then solubilised in 1% sodium deoxycholate. An aliquot of the solubilised membranes (10 μl) was blocked by adding 6× SDS/PAGE loading buffer [Input (washed)] and the remaining membrane fraction was submitted to immunoprecipitation using 1 µg of anti-NR1 antibody. The Input (washed) and the immunoprecipitated material [IP NR1 (washed)] were analysed by immunoblotting using the anti-calpain-1 antibody. The immunoreactive bands were quantified and the values are reported as the arithmetic mean ± SD of five different experiments.

To better investigate whether the association of HSP90 and calpain-1 with NMDAR could play a role in the activity of the protease, we analysed SK-N-BE cells which, as previously demonstrated express functional NMDAR [42, 43, 48]. As shown in Fig. 7A, in NR1 immunoprecipitation from resting SK-N-BE cells, we observed in addition to NR1, calpain-1 and high levels of HSP90, detectable amounts of NR2B. Conversely, neither calpain-2 nor calpastatin (both 110 and 140 kD forms) were associated with the NMDAR cluster. In order to assess a role for this resident calpain-1, we stimulated SK-N-BE cells with 500 µM NMDA for 30 min to increase the [Ca^2+^]_i_. As shown in Fig. 7B we observed that, the total amount of calpain-1 associated to the membranes was 3–4 folds increased as compared to untreated cells. Instead, the amount of calpain-1 detected following immunoprecipitation with anti-NR1, and thus complexed with NMDAR, remained substantially identical to that recovered in untreated cells. Moreover, the fraction of calpain-1 translocated at the membranes following cell Ca^2+^-loading can be washed out by 0.15 M NaCl solution, leaving the content of resident calpain-1 unmodified (Fig. 7B). Importantly, as we obtained similar results when cells were loaded directly with 1 μM calcium ionophore A23187 for 30 min (data not shown), the amount of resident calpain-1 at the NMDAR is independent of the calcium provenience. These data indicate for the first time that NMDAR cluster contains sites for calpain-1 association that are already saturated in basal conditions. The presence of resident calpain-1 in proximity of the Ca^2+^ channel could allow a rapid and limited activation of the protease that could be required for signal transduction processes.

We then investigated on the calpain targets associated with the NMDAR cluster possibly digested by resident calpain-1. SK-N-BE cells were stimulated as above and the proteins complexed with NR1 have been detected by specific antibodies. As shown in Table 1, both calcium ionophore and NMDA caused calpain-1 activation, as revealed by the digestion of several targets included in the NMDAR cluster. Specifically, HSP90 level was reduced up to 25–30%, whereas NR2B, a known calpain substrate unlike NR1 [39], was more than 60% decreased under both calpain activating conditions. Native nNOS (160 kD), also associated to the NMDAR cluster [48], was conservatively converted into the 130 kD active form [48], under both conditions. When the same experiments were carried out in the presence of synthetic calpain inhibitor-1 (CI-1) no proteolytic modifications occurred.

**Table 1:** Digestion of NMDA-R associated proteins following activation of resident calpain-1.

**Stimuli**	**HSP90**	**NR2B**	**160 kD nNOS**	**130 kD nNOS**
Vehicle	100 ± 7	100 ± 8	83 ± 8	18 ± 6
NMDA (500 μM)	85 ± 7	56 ± 7	44 ± 7	52 ± 6
NMDA (500 μM) + CI-1 (1 μM)	100 ± 8	103 ± 5	93 ± 7	10 ± 5
Ca^2+^-ionophore (1 μM)	75 ± 7	49 ± 7	38 ± 9	46 ± 7
Ca^2+^-ionophore (1 μM) + CI-1 (1 μM)	105 ± 5	106 ± 9	102 ± 7	8 ± 6

SK-N-BE cells were exposed to the indicated stimuli for 30 min at 37°C. The deoxycholate-soluble cell fraction was prepared as described in Methods and submitted to immunoprecipitation using anti-NR1 antibody. The immunoprecipitated material was analyzed by immunoblotting for the indicated proteins. The data shown are the quantification of the immunoreactive signals expressed as arbitrary units (means ± SD of five different experiments).

Based on these observations we suggest that resident calpain-1 at the NMDAR cluster could play a physiological role in controlling the Ca^2+^ influx by the digestion of NR2B. Furthermore, resident calpain-1 through the conversion of inactive nNOS to the active enzyme form, promotes an increased production of NO [43, 48].

To confirm that resident calpain-1 is responsible for such proteolytic events at the NMDAR cluster, we stimulated calpain activity directly in NR1-immunoprecipitated material. Aliquots of the immunoprecipitated NMDAR was suspended in 50 mM sodium borate buffer, pH 7.5 and incubated with 100 µM Ca^2+^ in the absence or presence of 1 µM CI-1. Following 30 min cell stimulation, the incubation was stopped by addition of SDS-PAGE loading buffer and samples were submitted to immunoblotting. As shown in Table 2, the calpain substrates were digested at an extent comparable to that observed in intact cells (see Table 1). CI-1 addition prevented completely the digestion, indicating that the amount of resident calpain-1 was sufficient to induce the proteolytic modifications detected in NMDAR complex.

**Table 2:** *In vitro* digestion of NMDA-R associated proteins by resident calpain-1 in isolated NR1 immunoprecipitates.

	**Resident calpain-1 substrates**			
**Conditions**	**HSP90**	**NR2B**	**160 kD nNOS**	**130 kD nNOS**
Control	100 ± 6	100 ± 8	80 ± 6	22 ± 6
Vehicle	100 ± 8	100 ± 8	86 ± 5	18 ± 7
CaCl_2_ (1 mM)	80 ± 8	27 ± 8	32 ± 7	58 ± 8
CaCl_2_ (1mM) + CI-1 (1 μM)	100 ± 5	96 ± 8	79 ± 7	24 ± 6

SK-N-BE cells were lysed and the deoxycholate-soluble cell fraction was prepared, as described in Methods. The samples were submitted to immunoprecipitation using anti-NR1 antibody. The immunoprecipitated material was left untreated (Control) or incubated in the indicated conditions for 30 min at 37°C and then analyzed by immunoblotting for the listed proteins. The data shown are the quantification of the immunoreactive signals expressed as arbitrary units (means ± SD of five different experiments).

**Figure 8:**
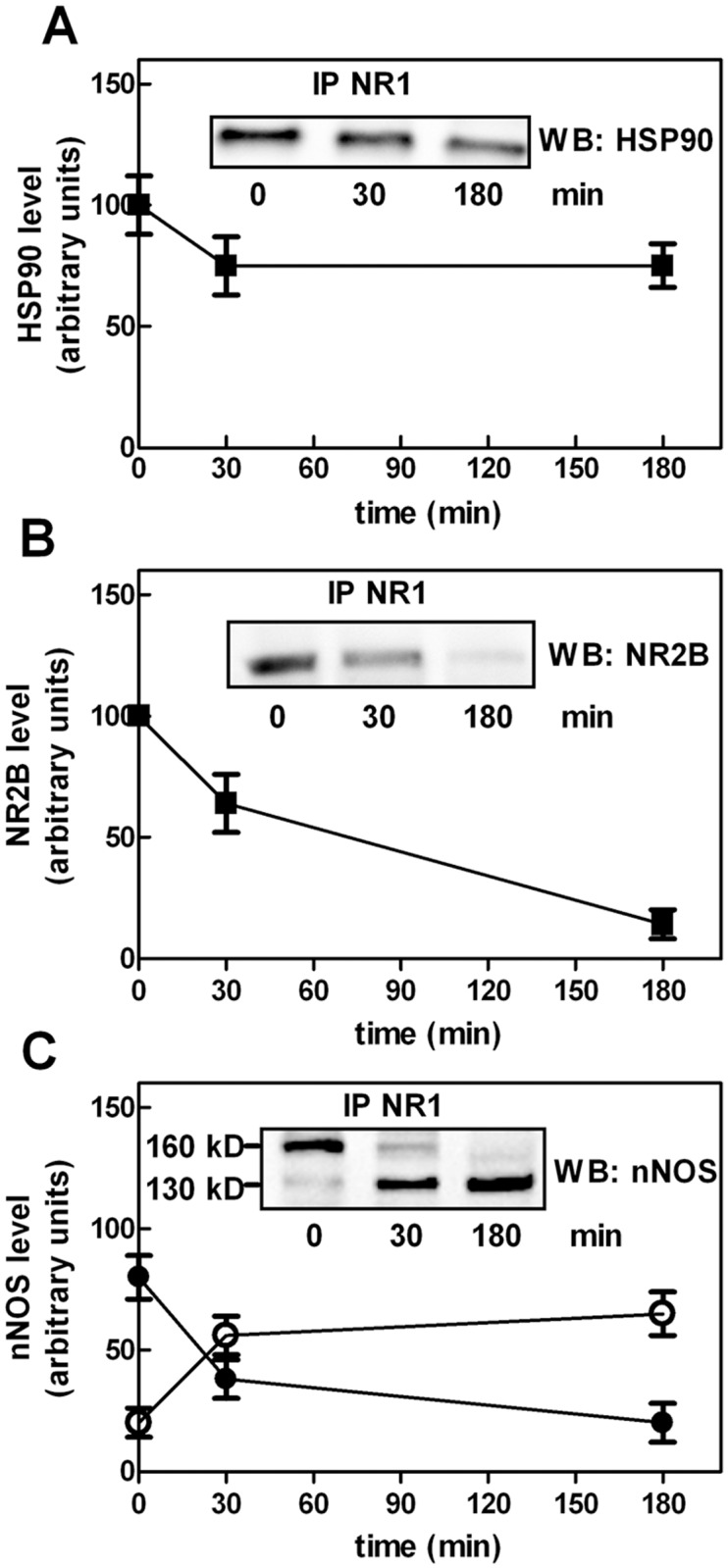
HSP90, NR2B, and nNOS levels in calcium-loaded SK-N-BE cells. SK-N-BE cells were treated with 500 µM NMDA for the indicated times. After treatment, the cells were lysed and submitted to immunoprecipitation using 1 µg of anti-NR1. The immunoprecipitated material (IP NR1) was submitted to immunoblotting to detect (A) HSP90, (B) NR2B, and (C) nNOS [160 kD (●) and 130 kD (○) forms]. The immunoreactive signals of five different experiments were quantified and are reported in the graphs as arithmetic mean ± SD. Insets: representative blots are shown.

We finally explored the kinetics of calpain-1 digestion in cells exposed to 500 µM NMDA for different times. As shown in Fig. 8, the digestion of NR2B and the conversion of native nNOS into the 130 kD form were the first proteolytic events catalyzed by resident calpain-1 whereas, HSP90 remained almost unaffected. The presence of a sufficient amount of HSP90 into the NMDAR cluster may be required to limit calpain-1 activity and maintain the proteolytic events in a physiological range.

## Discussion

It is currently considered that, following an increase in [Ca^2+^]_i_, activation of calpain occurs in coincidence with its translocation to sites preferentially located at the cell surface [1–6]. However, a “simple” Ca^2+^-induced translocation seems unable to assure the high degree of selectivity required for the physiological functions of calpain. At present no precise information are available on the existence of mechanisms driving calpain to specific sites of action.

We propose the involvement of HSP90, known to assist a large number of proteins involved in cell signalling [49, 50], in the intracellular trafficking of calpain-1. Although HSP90 shows a low degree of specificity, in our conditions it is highly selective distinguishing calpain-1 from calpain-2.

HSP90-calpain-1 interaction occurs through the formation of a complex with a molecular mass of approximately 300 kD that results from the association of an equal number of molecules of each protein. The chaperone bound to calpain-1 is protected from the digestion by the protease although the active site is not affected by this interaction since the catalytic cleft remains accessible to free HSP90 molecules.

In the present paper we suggest that HSP90 is involved in the dynamic activation of calpain. We here observed that HSP90 competes with calpastatin for binding to calpain-1. Since it is known that in resting cells calpastatin is preferentially present in aggregates [29], cytosolic calpain-1 is predominantly associated to HSP90. When [Ca^2+^]_i_ is increased and calpastatin diffuses in the cytosol, calpain-1, interacting with the inhibitor, can form two binary complexes. In the first one, constituted by HSP90 and calpain-1, the protease is still activable whereas, in the second one, composed by calpastatin and calpain-1, the protease is inactive and unable to reach the activation sites.

Our findings suggest that HSP90 has a role in the dynamic activation of cytosolic calpain-1 during transient and moderate elevations in [Ca^2+^]_i_. In these conditions HSP90 associated with calpain-1 maintains the protease in a low active state without affecting the Ca^2+^-dependent translocation of the enzyme. We have also established that in resting cells calpain-1 is present together with HSP90 as an integral component of the NMDAR cluster. This association is very specific since calpain-2 was never present at the NMDAR complex, and it is stable, as the amount of calpain-1 associated with the complex remains unaffected also following cell Ca^2+^-loading. Resident calpain-1 could be functionally important as it may be promptly activated following the stimulation of NMDAR. The local and rapid increase in [Ca^2+^] overcomes not only the high calcium requirement for calpain activation but also the effect exerted by HSP90 on the protease. The efficacy of calpain-1 associated with NMDAR derives also from the fact that the protease is in proximity of its targets. The rapid calpain-1-mediated digestion of NR2B, which is known to be involved in the control of the Ca^2+^ influx, could represent a possible defense mechanism against calcium overload [51]. We have demonstrated that the digestion of calpain targets at the NMDAR is mediated exclusively by resident calpain-1. This important observation, first obtained in intact cells, has been confirmed by activating directly the calpain-1 inserted in isolated NMDAR clusters. At this regard it has been recently reported [14] a relationship between the localization of both calpain-1 and-2 and their functional role in nervous cells.

In conclusion, the HSP90 that specifically interacts and associates with calpain-1, assists the protease by increasing its Ca^2+^-requirement and by regulating its recruitment at selective cell sites. We demonstrate for the first time that, in basal conditions, calpain-1 is stably localized at NMDAR cluster in an integrated form together with HSP90. Both HSP90 and calpain-1 could be responsible for those NMDAR physiological processes occurring following activation of this glutamate-gated ionotropic receptor.

It is tempting to speculate that HSP90 could be involved in targeting proper amounts of calpain-1 at the NMDAR multiprotein complex. This resident calpain-1 may regulate the transduction signalling elicited by Ca^2+^ influx across the channel pore.

We have previously demonstrated that calpain-1 and HSP90 are also associated with the cystic fibrosis transmembrane conductance regulator (CFTR) protein clusters [52]. Several members of this complex are digested by calpain-1 as observed in the NMDAR clusters. Since many calpain targets [13–18] are associated with channels/receptors, we can propose that calpain-1-HSP90 interplay may have a general involvement in the regulation and function of these multi-protein structures.
